# Unraveling the Intricate Roles of Exosomes in Cardiovascular Diseases: A Comprehensive Review of Physiological Significance and Pathological Implications

**DOI:** 10.3390/ijms242115677

**Published:** 2023-10-27

**Authors:** Shuai Zhang, Yu Yang, Xinchen Lv, Wendong Liu, Shaohua Zhu, Ying Wang, Hongfei Xu

**Affiliations:** Department of Forensic Medicine, School of Basic Medicine and Biological Sciences, Soochow University, Suzhou 215123, China; 15225377016@163.com (S.Z.); 20224221089@stu.suda.edu.cn (Y.Y.); l18733160301@126.com (X.L.); lwdyyds@163.com (W.L.); zhushaohua@suda.edu.cn (S.Z.)

**Keywords:** exosome function, cardiovascular disease, intracellular communication, biomarkers, exosome therapeutics

## Abstract

Exosomes, as potent intercellular communication tools, have garnered significant attention due to their unique cargo-carrying capabilities, which enable them to influence diverse physiological and pathological functions. Extensive research has illuminated the biogenesis, secretion, and functions of exosomes. These vesicles are secreted by cells in different states, exerting either protective or harmful biological functions. Emerging evidence highlights their role in cardiovascular disease (CVD) by mediating comprehensive interactions among diverse cell types. This review delves into the significant impacts of exosomes on CVD under stress and disease conditions, including coronary artery disease (CAD), myocardial infarction, heart failure, and other cardiomyopathies. Focusing on the cellular signaling and mechanisms, we explore how exosomes mediate multifaceted interactions, particularly contributing to endothelial dysfunction, oxidative stress, and apoptosis in CVD pathogenesis. Additionally, exosomes show great promise as biomarkers, reflecting differential expressions of NcRNAs (miRNAs, lncRNAs, and circRNAs), and as therapeutic carriers for targeted CVD treatment. However, the specific regulatory mechanisms governing exosomes in CVD remain incomplete, necessitating further exploration of their characteristics and roles in various CVD-related contexts. This comprehensive review aims to provide novel insights into the biological implications of exosomes in CVD and offer innovative perspectives on the diagnosis and treatment of CVD.

## 1. Introduction

The cardiovascular system constitutes a dynamic network of diverse cell types that collectively facilitate blood circulation throughout the body [1,2]. In the face of the challenges posed by a complex survival environment, a complex and finely tuned system to facilitate cell-cell homeostasis mechanisms is required to ensure that the beating heart normally and efficiently pumps blood into the vessels, thus finely controlling perfusion and fluid balance. Consequently, to sustain blood supply to the heart and ensure optimal myocardial function, effective communication among various cell types within the heart, such as cardiomyocytes, smooth muscle cells, endothelial cells (ECs), fibroblasts, mast cells, immune-system related cells, and other connective tissue cells, is indispensable. This intricate communication framework plays a pivotal role in coordinating information exchange, orchestrating the operation of multi-cellular systems, and maintaining overall cardiovascular health [3,4,5,6].

Cardiovascular disease (CVD) stands as the foremost cause of global mortality, accounting for approximately 25 percent of all deaths and imposing a substantial economic burden on society and the healthcare system [7]. Manifesting as vascular and cardiac disorders, CVD is intricately linked to progressive aging and primarily involves the arteries and heart [8,9,10]. This progressive biological process significantly affects the cardiovascular system, ultimately heightening the risk of conditions such as atherosclerosis, hypertension, myocardial infarction, and stroke [10,11]. The primary clinical events underlying CVD are predominantly of vascular origin [12]. Extensive evidence underscores the important role of inflammation, particularly ECs dysfunction, in CVD pathogenesis [13]. ECs with an inflammatory phenotype cause vascular inflammation, thereby leading to cardiac dysfunction [14]. Despite advances in cardiology aimed at optimizing coronary flow restoration and improving survival rates, outcomes following ischemia/reperfusion (IR) injury remain unsatisfactory. CVD often progresses to hypertrophy and heart failure, resulting in substantial mortality rates [15,16]. Therefore, the development of early diagnostic or treatment strategies assumes paramount importance in reducing CVD-related mortality.

Exosomes, their generation, and their function mechanisms in multicellular organisms span from physiological tissue regulation to pathogenic injury and organ remodeling. The field has been stimulated by numerous studies highlighting the correlation between circulating exosomes and the risk and severity of various diseases, including CVD, diabetes, kidney disease, and cancer [17,18,19,20]. Exosomes, deriving from diverse cell types, including those within the cardiovascular system, exist in various body fluids, including blood, urine, saliva, amniotic fluid, and breast milk [21]. Recently, exosomes, which are naturally released by mammalian cells, have emerged as pivotal tools for intercellular cardiac communication [22,23,24], significantly influencing vascular integrity and CVD [25,26]. Functioning as cargo carriers, exosomes encapsulate complex substances [27], with cargo composition varying according to the originating cell type and its “health status”. Consequently, exosomes yield disparate functional outcomes upon incorporation into recipient cells. These vesicles mediate multifaceted processes and pathways, underpinning both the normal cardiovascular physiology, such as heart development and myocardial angiogenesis [28], and pathophysiological conditions, including cardiac hypertrophy [29], atherogenesis [30], heart failure [31], and hypertension [32]. Notably, cardiac exosomes can induce endothelial dysfunction [33], oxidative stress [34], and eventual apoptosis [35], forming key pathways in the pathogenesis of CVD. However, comprehensive studies elucidating exosome phenotypes in CVD patients remain scarce, and the precise role of exosomes in CVD pathophysiology remains enigmatic.

Questions surrounding exosome function have focused on understanding the fate of their components and the phenotypic and molecular alterations that they induce in recipient cells in cell culture systems. A growing body of evidence suggests that exosomes may facilitate the transfer of non-coding RNAs (NcRNAs), such as miRNAs and lncRNAs, to recipient cells, thereby triggering gene expression and signaling pathways that drive cell communication and phenotypic alterations [36,37]. Additionally, exosomes have garnered attention as potential therapeutic targets [38], drug delivery agents [39], and tools for diverse biomedical applications [40]. Their efficiency in delivery and functional versatility underscore their potential. In this review, our focus centers on exosomes and their implications in cardiac pathophysiology. Specifically, we delve into exosome size, biogenesis, secretion, and functionality. Moreover, we provide a comprehensive overview of the dual roles of exosomes in several major cardiovascular pathologies, including coronary artery disease (CAD), myocardial infarction, heart failure, and other cardiomyopathies. Lastly, we explore the diagnostic, prognostic, and therapeutic potential of exosomes in CVD.

## 2. Overview of Exosomes

### 2.1. Exosome Size and Biogenesis

Until recently, the role of extracellular vesicles (EVs), particularly exosomes, in biogenesis, and local and remote cell-cell signaling has not been fully recognized. Various types of membrane vesicles produced by cells include apoptotic bodies, microvesicles (MVs), and exosomes [41]. These vesicles can be distinguished by their size, route of origin, and contents. Exosomes, the smallest EVs released by donor cells, have diameter ranging from 30–150 nm in diameter. In contrast, MVs and apoptotic bodies are 100–1000 nm and 50–5000 nm in diameter, respectively, both belonging to the subclass of EVs [42,43,44].

**Figure 1:**
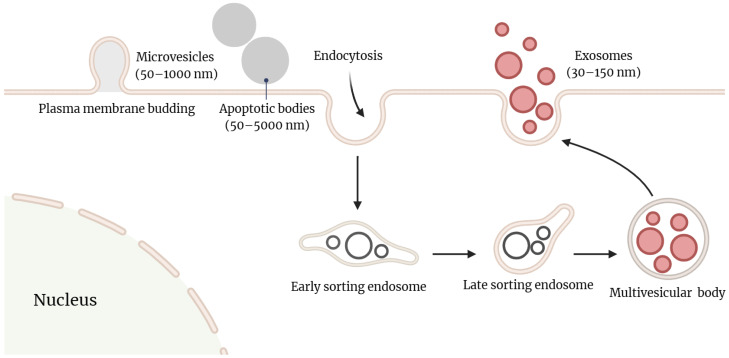
The three major categories of EVs: microvesicles, apoptotic bodies, and exosomes. Microvesicles are released through plasma membrane budding and have a size range of 50 nm to 1000 nm. Apoptotic bodies are released by cells undergoing apoptosis as blebs into the extracellular space and have diameter ranging from 50–5000 nm. Exosomes originate in the endosomal pathway through the formation of early-sorting endosomes, late-sorting endosomes, and eventually multivesicular bodies. They are released when multivesicular bodies fuse with the plasma membrane and range in size from 30 to 150 nm. This figure was created with BioRender.com.

Unlike apoptotic bodies, which form from vesicles of dead or dying cells, and MVs, which bud outward from the plasma membrane, the biogenesis of exosomes is a complex and tightly regulated process [45,46]. Initially, early-sorting endosomes (ESEs) are formed through plasma membrane invaginations. These ESEs, along with their cytoplasmic contents, are then internalized into late-sorting endosomes (LSEs), eventually forming multivesicular bodies (MVBs) containing numerous intraluminal vesicles (ILVs) that will become exosomes. Subsequently, MVBs can either fuse with lysosomes or autophagosomes for degradation or merge with the plasma membrane to release the enclosed ILVs as exosomes (Figure 1) [47,48,49]. Additionally, exosomes contain unique biomarkers that distinguish them from MVs and apoptotic bodies, such as tetraspanins (e.g., CD63, CD9, and CD81), TSG101 (tumor susceptibility gene 101), syntenin-1, ALIX (apoptosis-linked gene 2-interacting protein X), and ceramides. These biomarkers are enriched in exosomes and play role in the origin and biogenesis of exosomes [50,51]. However, the specific roles and functions of these proteins in exosome biogenesis require further in-depth exploration.

Initially, when exosomes were first identified in mature sheep reticulocytes in the late 1980s, they were thought to be cell fragments with no significant impact on neighboring cells [52]. However, recent evidence suggests that these nanoscale EVs are functional carriers for cargo transport [53]. Exosomes can transport a variety of molecules, including nucleic acids, proteins, lipids, amino acids, and metabolites, which reflect their cellular origin and have a profound influence on the phenotype of recipient cells [54,55]. They can shuttle between cells in an autocrine, paracrine, or endocrine manner, thereby influencing the state of the recipient cells. It is becoming increasingly clear that exosomes are important players in both the normal physiology and pathophysiology of cells.

### 2.2. Secretion of Exosomes

**Figure 2:**
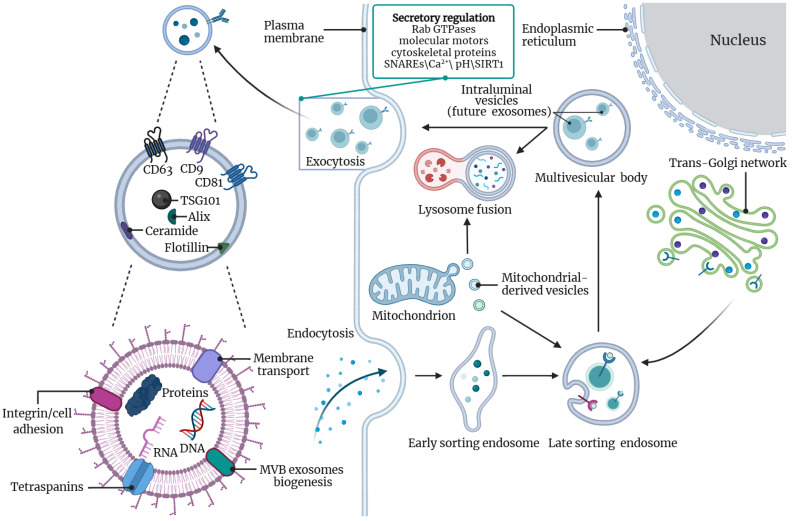
Biogenesis, secretion, and identification of exosomes. Fluid and extracellular components, such as proteins, lipids, metabolites, small molecules, and ions, can enter cells through endocytosis and plasma membrane invagination, along with cell surface proteins. The plasma membrane bud formed on the side of the cell cavity shows outward-to-inward orientation of the plasma membrane. This budding process leads to the formation of early-sorting endosomes or possible fusion of the bud with early-sorting endosomes preformed by the trans-Golgi network (TGN) and mitochondrial-derived vesicles. The second invagination of late-sorting endosomes leads to the production of intraluminal vesicles, further modifying the cargo of future exosomes and allowing the entry of cytoplasmic components into the newly formed intraluminal vesicles. As part of intraluminal vesicles formation, proteins originally present on the cell surface can be clearly distributed within the intraluminal vesicles. Depending on the extent of invagination, this process can produce intraluminal vesicles of varying sizes and contents. Late-sorting endosomes produce multivesicular bodies with a well-defined set of intraluminal vesicles (future exosomes). Multivesicular bodies can either fuse with lysosomes for degradation or, if following a different trajectory, be transported to the plasma membrane via the cytoskeleton and microtubule networks. Exocytosis results in the release of exosomes, which possess lipid bilayers similar to the plasma membrane. Exosome biogenesis is associated with factors such as Ca^2+^, pH, SIRT1, and proteins like Rab GTPases and ESCRT, as discussed in the text. Additionally, several proteins are commonly used as exosome markers, including CD9, CD81, CD63, flotillin, TSG101, ceramides, and Alix. Exosome surface proteins encompass tetraspanins, integrins, membrane transport proteins, and more. Exosomes contain various cell surface proteins, intracellular proteins, RNA, and DNA. This figure was created with BioRender.com.

Exosome secretion is a vital cellular mechanism observed both in vivo and in vitro (Figure 2) [56,57]. This process encompasses two major modes: the endosomal sorting complex required for transport (ESCRT)-dependent and ESCRT-independent pathways [58]. ESCRT, which plays a key role in cell membrane repair, is particularly relevant to exosome biology. The release of exosomes occurs subsequent to the fusion of MVBs with the plasma membrane, with ESCRT playing an essential role in exosome formation [59,60]. Components within the ESCRT act as important regulators during the generation of MVBs and ILVs [61,62]. Notably, ESCRT depletion has been shown to inhibit exosome biogenesis [61]. The involvement of ESCRT in exosome biogenesis occurs in stages [62,63]. Initially, ESCRT-0 targets the ESEs. This is followed by activation of ESCRT-I and -II, which are involved in membrane deformation and budding, thereby isolating the parent cell cargo. ESCRT-III then drives vesicle scission. Moreover, independent of ESCRT, alternative mechanisms have also been described. In certain cellular systems, ESCRT-independent ILVs formation in MVBs requires lipid rafts and ceramide [64,65,66], and/or aggregation of tetraspanins [67,68,69]. Among tetraspanins, CD63, which is particularly enriched intracellular and is primarily confined to LSEs and lysosomes [70], plays a role in ESCRT-independent ILVs formation, thereby regulating exosome secretion.

Exosome secretion is a multifaceted process influenced by various factors, including Ras-related protein Rab GTPases, molecular motors, cytoskeletal proteins, and SNAREs [soluble Nethylmaleimide-sensitive factor (NSF) attachment protein receptors] complex proteins. Intracellular Ca^2+^ levels also impact exosome secretion, with elevated Ca^2+^ levels leading to increased exosome release. Additionally, both intracellular and extracellular pH gradients are known to influence this process [71,72]. Recent studies have shed light on the role of silencing information regulator factor 1 (SIRT1) expression in exosome release [73]. SIRT1, a histone deacetylase, plays an important role in various physiological and pathological processes, including cell metabolism, cell survival, cell senescence, DNA repair, cell proliferation, differentiation, apoptosis, and inflammation [74,75]. Reduced SIRT1 expression restricts the expression of a specific subunit of vacuolar H+ ATPase (V-ATPase), which is responsible for proper lysosomes acidification and protein degradation. This restriction can result in a decreased number and increased size of degraded MVBs prior to fusion with the plasma membrane, ultimately leading to enhanced exosome secretion from the cells [76]. Moreover, it is worth noting that mitochondrial-derived vesicles (MDVs) have been suggested as one of the sources of exosome release. In the inflammatory state, the release of MDVs is significantly increased [77,78,79,80,81,82]. During this process, mitochondrial cargo is transported to the endolysosome system for mitochondrial quality control (MQC) [83]. The Syntaxin-17-SNAP29-VAMP7 terpolymer facilitates the delivery and fusion of MDVs to endosomes/lysosomes [84]. Importantly, in addition to being transported to lysosomes for degradation, some MDVs specifically reach endosomes to form MVBs and are subsequently released as exosomes into the extracellular environment [85]. Proteomic studies have demonstrated that up to 10% of exosomes originate from mitochondria [86,87]. These findings highlight the significance of MDVs as contributors to exosome secretion and provide novel insights into the mechanisms governing exosome production and release.

### 2.3. Function of Exosomes

In recent years, the study of exosomes as important mediators of cell communication has emerged endlessly [88,89]. Exosomes facilitate communication with recipient cells through various mechanisms, including ligand binding directly to the receptor to activate downstream signaling, direct membrane fusion resulting in the release of exosome contents into the recipient cells, and internalization of exosomes by the recipient cells through endocytosis (including phagocytosis, macrophage, or receptor-mediated endocytosis) [90]. Exosomal proteins can promptly stimulate recipient cells upon contact, thereby regulating cellular behavior [91]. A recent unexpected discovery has unveiled that exosomes contain double-stranded DNA, mRNA, and NcRNAs (including miRNAs, IncRNAs, and circRNAs) [92,93,94,95]. Increasing evidence suggests that the proteins and NcRNAs carried by exosomes can be inserted into recipient cells, inducing transient or long-term phenotypic changes [29,91,96,97]. These contents within exosomes are encapsulated by lipid or lipoprotein complexes, providing protection from degradation during transportation. Exosomes are involved in a variety of regulatory mechanisms [98], encompassing cell signaling, cell differentiation, immune regulation, substance metabolism, gene regulation, and tumor cell growth [99,100]. These functional molecules exhibit relative stability and can reflect the cellular origin, undergoing changes in response to variations in proteins and metabolites within the cells. Exosomes derived from different cell types exhibit pleiotropic biological activities, which can be either protective or deleterious, depending largely on the origin and current state of the cells [101]. This highlights the potential of exosome contents exchange between cells as an effective mode of intercellular communication. Specific cellular components contained within exosomes possess distinct functions and targeting capabilities, suggesting a role in regulation of intercellular communication [40].

The biological significance of exosomes in diseases is still emerging, with a substantial increase in studies exploring their utility in various pathological diagnosis and treatments. Exploiting the diverse cargo of exosomes provides a multifaceted diagnostic window for disease detection and monitoring. Diseases that have garnered significant attention for the diagnostic application of exosomes include CVD [102,103], central nervous system diseases [104,105], and cancer [106,107]. Under pathological conditions, cells release a plethora of exosomes containing diverse substances that significantly contribute to the progression of diseases. It is now understood that sorting of proteins and RNA in exosomes is specifically regulated by various pathophysiological stress stimuli and disease conditions [95,108]. This allows cells to generate tailored exosomes with distinct functional characteristics that reflect their parent cell state, with stress or disease conditions manifesting in the exosome contents. Consequently, the circulation of exosomes can be used as biomarkers for diagnostic and prognostic strategies in the context of pathological processes of CVD.

**Figure 3:**
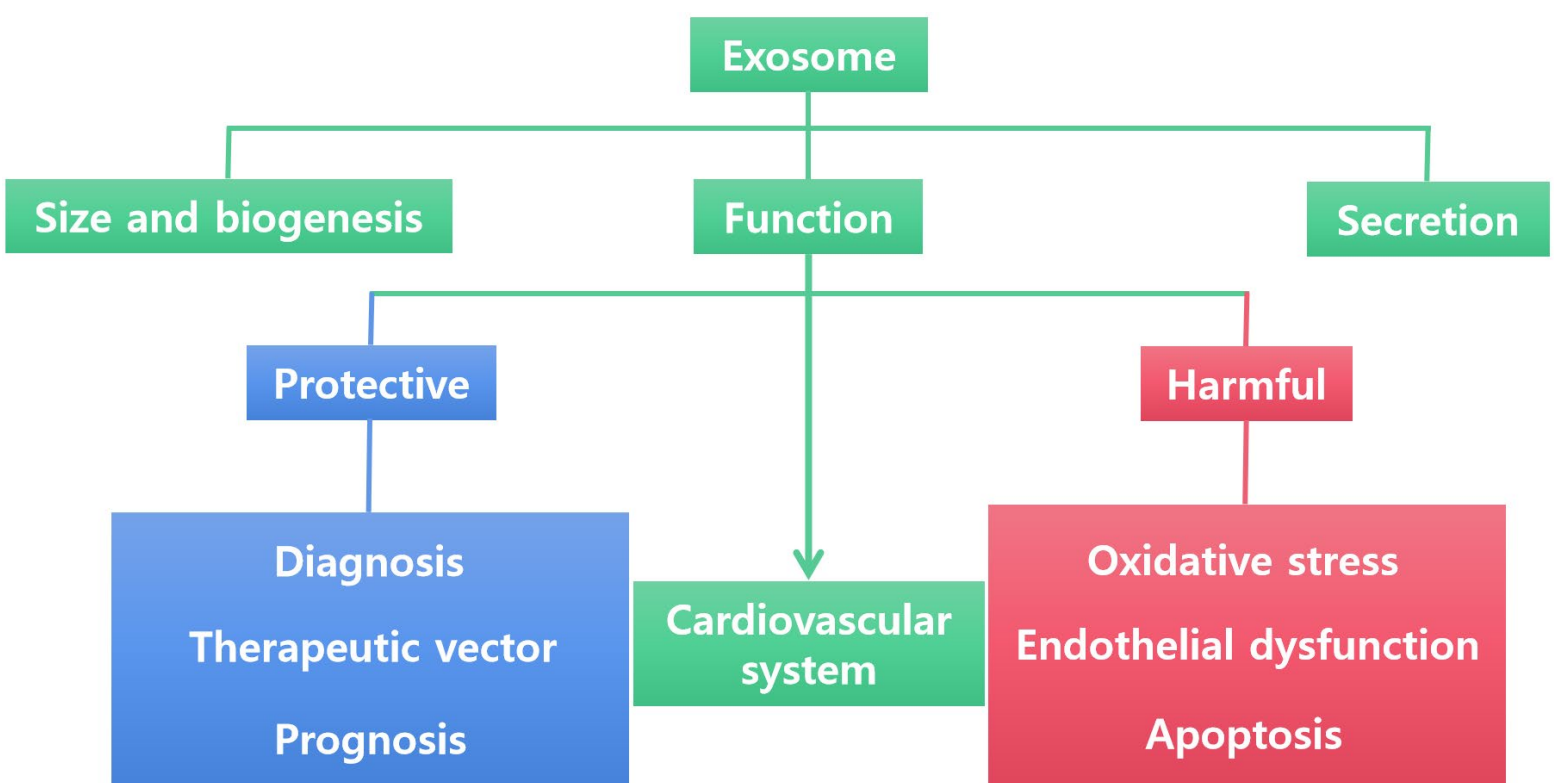
Biogenesis and secretion of exosomes and their contrasting biological functions in the cardiovascular system. The biogenesis and secretion of exosomes can play either a protective role (in diagnosis, as therapeutic vector, and in prognostic evaluations) or a harmful role (by inducing oxidative stress, endothelial dysfunction, and apoptosis) in the context of the cardiovascular system.

Since the first isolation of exosomes from cardiomyocytes cultured in vitro in 2007, the potential role of exosomes as diagnostic biomarkers or prognostic identifiers for CVD has gained considerable attention (Figure 3). Meanwhile, exosomes have been shown to mediate communication between various cardiovascular cells, including ECs and smooth muscle cells [109], cardiomyocytes and ECs [97], and fibroblasts and cardiomyocytes [29]. Over the past decade, exosomes as carriers of miRNAs and proteins in the field of CVD have been extensively studied [110], suggesting that exosomes may play a key role in the diagnosis and prognosis of CVD. The cargo contained within exosomes and the communication among cardiovascular cells are believed to be involved in the development of CVD [111,112]. It is becoming increasingly evident that both local and long-distance intercellular communication contribute to the maintenance of normal cardiac homeostasis and responses to hypertrophic stimuli [5,113,114]. However, exosomes released by cells affected by CVD can also exert opposite effects and further exacerbate disease progression [115,116,117]. Therefore, the multifaceted functions of exosomes in cardiovascular pathophysiology hold significant promise for a wide range of applications, which will be of great significance in exploring the pathogenesis of CVD.

## 3. Exosomes in Cardiovascular Disease

### 3.1. Exosomes in Coronary Artery Disease

CAD is a prevalent disease closely linked to increased cardiovascular morbidity and mortality, imposing a substantial economic burden on the healthcare system [118,119]. Advances in our comprehension of atherosclerosis mechanisms, the development of antiplatelet and statin medications, and progress in coronary interventional procedures have significantly improved clinical outcomes for CAD patients [120]. However, individuals with CAD still face risk such as stent restenosis, adverse cardiac remodeling, and IR injury. Atherosclerosis, primarily characterized by coronary artery plaque formation, underlies CAD pathogenesis [121]. This intricate process involves oxidative stress, endothelial dysfunction, and inflammation as pivotal contributors [122,123,124,125]. Monocytes or macrophages accumulation within blood vessel walls, resulting in the production of pro-inflammatory cytokines, is a fundamental event in atherosclerosis development [126]. Notably, exosomes released by primary human monocytes can be internalized by ECs, inducing endothelial dysfunction through the TLR4 and NF-κB pathways [127]. Another study [128] reported that lipopolysaccharide (LPS)-activated exosomes released by macrophages impact gene expression and differentiation of adipocytes, potentially playing a key role in atherosclerosis.

**Figure 4:**
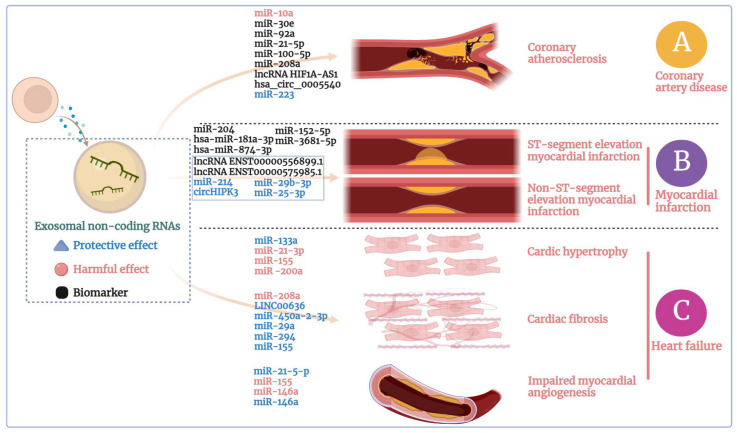
Different functions of exosomal NcRNAs in CVD. Exosomal NcRNAs can have protective effects (blue) or harmful effects (red) in: (**A**) coronary artery disease, especially in the context of in coronary atherosclerosis, (**B**) myocardial infarction, encompassing both ST-segment elevation MI (TEMI) and non-ST-segment elevation MI (NSTEMI), and (**C**) heart failure scenarios such as cardiac hypertrophy, cardiac fibrosis, and myocardial angiogenesis. Addiontially, these exosomal NcRNAs can also serve as biomarkers (black). This figure was created with BioRender.com.

Exosomes derived from CAD patients have been shown to inflict endothelial injury and inflammation, contributing to endothelial dysfunction [33]. Importantly, exosomes, as natural carriers of RNA, have received significant attention in recent years for their pro-inflammatory roles in early atherosclerosis stages [129,130]. NcRNAs play important roles in the development of CAD through different mechanisms (Figure 4A) [131]. Of note, miRNAs encapsulated within exosomes [132] can be secreted by a variety of cell types, including immune cells (T and B cells) [133], stem cells [134,135], peripheral blood cells (lymphocytes, monocytes, macrophages, and platelets) [136], and adipocytes. Exosomes originating from different cell types contribute significantly to inflammation [130] and participate in all stages of atherosclerosis [137]. Moreover, exosomes released by a variety of cell types and platelets carry valuable biological information about CAD onset and progression [138]. They engage in both short- and long-range intercellular communication within the cardiovascular system through the transfer of miRNAs and other mediators [139]. Recent research has revealed that cardiac exosomes can activate inflammation via the TNF-α-mediated NF-κB pathway by transporting miR-10a [138,140]. Additionally, miR-30e and miR-92a are overexpressed in the plasma exosomes of patients with coronary artery atherosclerosis and hold promise as novel diagnostic biomarkers [141,142]. There is also evidence linking the levels of miR-21-5p and miR-100-5p in exosomes to both age and the severity of CAD [143]. Clinical studies have shown elevated serum exosomal miR-208a expression in patients with acute CAD compared to healthy subjects [144]. Furthermore, exosome concentrations and the expression level of exosomal lncRNA HIF1A-AS1 in atherosclerosis patients were significantly higher than in healthy individuals suggesting potential biomarkers for prognosis prediction [145]. In these CAD patients, the mortality is significantly increased during one-year follow-up. Another study found a significant increase in plasma and cardiac exosome miRNAs 48 h after coronary artery bypass surgery [146]. These exosomes and their contained miRNAs are positively correlated with high-sensitivity cardiac troponin I (hs-cTnI), a cardiac biomarker. Moreover, compared with miRNAs and lncRNAs, circRNAs are structurally stable and have long half-lives without degradation in exosomes, making them reliable biomarkers [147,148]. Analysis of circRNA expression in plasma exosomes in patients with CAD found plasma exosomal hsa_circ_0005540, which can be used as a promising diagnostic biomarker of CAD [149].

Intriguingly, exosomes may also exert protective effects in cardiovascular health. Given their natural role as carriers of bioactive signaling molecules, exosomes have garnered attention as potential biomarkers and therapeutic agents. Studies involving THP-1 and human embryonic kidney cells have shown that HSP27-laden exosomes significantly stimulate NF-κB activation and IL-10 release, suggesting that HSP27 could be an essential exosome cargo with beneficial anti-inflammatory effects [150]. In addition, artificially benign modifications or cardioprotective medications can induce cells to produce protective exosomes. Molecularly engineered M2 macrophage-derived exosomes, further electroporated with 5-aminolevulpiate hexyl ester, have been reported to alleviate inflammation by promoting the release of anti-inflammatory cytokines [121]. Paeonol has been reported to inhibit atherosclerosis by upregulating miR-223 expression in monocyte exosomes, thereby inhibiting the STAT3 pathway [151]. NcRNAs within exosomes hold immense promise as biomarkers for CAD detection and are expected to provide crucial insight for inhibiting atherosclerosis, which may have breakthrough significance for developing new targets for the prevention and treatment of CAD.

### 3.2. Exosomes in Myocardial Infarction

Myocardial infarction is a detrimental cardiac event resulting from the transient or sustained occlusion of a coronary artery, leading to myocardial necrosis and loss of cardiomyocytes [152]. Acute myocardial infarction represents one of the most severe forms of coronary heart disease (CHD) [153]. The complete occlusion of blood vessels, consequent to the lack of blood flow perfusion in the myocardium, can precipitate severe heart failure, fatal arrhythmia, cardiogenic shock, and even cardiac arrest, posing a severe risk to patient lives [154]. Current clinical practice indicate that timely reperfusion therapy has significantly improved outcomes by restoring myocardial blood flow and perfusion [155,156]. However, inevitable reperfusion injury and dysregulated immune responses often lead to detrimental ventricular remodeling and increased risk of heart failure progression [157]. Therefore, early and accurate diagnosis is paramount for improving clinical outcomes and reduce mortality.

Acute myocardial infarction categorizes into ST-segment elevation myocardial infarction (STEMI) and non-ST-segment elevation myocardial infarction (NSTEMI) [153]. STEMI typically arises from coronary atherosclerosis, characterized by a prominent presence of red blood cells and red fibrin thrombi, leading to a complete disruption of coronary blood flow [158]. Notably, approximately half of the STEMI patients might develop symptoms of heart failure between 3–6 months after infarction [159]. Early diagnosis and immediate reperfusion are pivotal for mitigating the impact of myocardial infarction and infarct size, thereby reducing potential complications and heart failure after STEMI [160]. Relatively, NSTEMI is often the result of unstable plaque formations, which lead to the partial occlusion of the coronary artery, albeit with minimal blood flow [161,162]. Although Electrocardiogram (ECG) indications of ST-segment elevation serve as sensitive and specific signs of Total coronary Occlusion (TO) in STEMI patients, TO is detected in merely 25.5–34% of NSTEMI patients [153]. Patients exhibiting TO often face delayed diagnoses, restricted access to intervention, and increased complications and mortality rates. Therefore, methods for rapidly diagnosing STEMI and NSTEMI onset are clinically valuable. Such methodologies can potentially reduce the mortality rates and improve patient prognosis [161,163].

Within the myocardial infarction context, exosomes facilitate both local and remote microcommunication among recipient cells (Figure 4B) [23,164]. It is worth noting that the content [165] and quantity [146,166] of circulating exosomes undergo significant alterations during this process. This suggests that the compromised myocardium releases specific exosomes into bodily fluids. The study found that the expression of lncRNAs ENST00000556899.1 and ENST00000575985.1 in circulating exosomes in patients with acute myocardial infarction was significantly higher than that in healthy individuals, which can be used as potential biomarkers [167]. Research has also shown that serum exosomal NEAT1, miR-204, and MMP-9 serve as potent biomarkers for acute STEMI diagnosis [168]. The prevalent adoption of medical treatments and revascularization techniques have been linked to increased early survival rates in STEMI patients [169,170]. However, limited studies have ventured into forecasting biochemical indicators associated with long-term myocardial remodeling [171]. Exosomes offer a robust evaluation mechanism in this regard. Research has elucidated that exosomes sourced from STEMI-diagnosed patients who underwent coronary angioplasty notably reduce cardiomyocyte viability [172]. Comparative studies between healthy subjects and patients 3–6 months after STEMI revealed the differential regulation of 28 miRNAs (upregulated) and 49 miRNAs (downregulated). Among these, the two most underexpressed miRNAs in heart-related exosomes are hsa-miR-181a-3p and hsa-miR-874-3p [173]. Exosomal miRNAs analysis, when combined with Real-Time Three-Dimensional Spot Tracking Echocardiography (RT3D-STE), are expected to evolve into a nuanced diagnostic system for acute myocardial infarction. Such an advancement would proficiently differentiate STEMI from NSTEMI, thus ensuring detailed and precise diagnostic outcomes [153]. The circulating heart-specific exosomal miR-152-5p and miR-3681-5p are projected to be pivotal biomarkers for the early diagnosis of STEMI and NSTEMI. Therefore, exosomal miRNAs hold immense potential as biomarkers for the early diagnosis and prognositic assessment of STEMI or NSTEMI. Notwithstanding, research elucidating the relationship between STEMI or NSTEMI and exosomes remains nascent. It is imperative to discern the key mechanisms underlying cargo classification and functional regulation, which encompasses the unique packaging of miRNAs and concomitant gene expression pathways.

On the other hand, exosomes originating from normative or benign cells have been implicated in the onset and progression of cardiac outcomes as well as repair processes subsequent to myocardial infarction. Exosomes sourced from healthy individuals confer protection ischemic myocardium through the conveyance of endogenous defensive signals, including the cardioprotective protein HSP70 [174]. The cardioprotective miR-214 has been exhibited an upregulation in the heart after ischemia and is secreted by exosomes from human ECs [175,176]. Further research has found that cardiomyocyte exosomal circHIPK3 might emerge as a therapeutic target for IR [177]. The hypoxia-induced upregulation of circHIPK3 in cardiomyocyte exosomes promotes angiogenesis and limits myocardial infarction area. This phenomenon occurs partly through the miR-29a/VEGFA axis, thereby safeguarding myocardial functionality and ECs integrity after myocardial infarction, offering myocardial protection. In the peri-infarct area, the paracrine activity of exosomes derived from unharmed myocardium might orchestrate cardiomyocytes reprogramming by the transference of molecules such as RNAs and peptides, rescuing the peri-infarct area. This subsequently attenuates necrosis, inflammation, apoptosis, remodeling, and fibrosis [178]. Predominant animal studies have administered stem cell-derived exosomes via intramyocardial delivery. During myocardial infarction episodes, exosomes released by cardiac progenitor [179] or embryonic stem cells [180] modulate cardiac regeneration and remodeling. Under hypoxic conditions, cardiac progenitor cells curtail cardiac fibrosis after myocardial infarction, impede cardiomyocyte apoptosis, and increase angiogenesis or cardiac output by transporting exosomes laden with anti-fibrotic miRNAs to fibroblasts [181]. Exosomes secreted by bone marrow MSCs transport miR-29b-3p, which targets ADAMTS16, ameliorating angiogenesis and ventricular remodeling in myocardial infarction-afflicted rats [182]. Exosomal miR-25-3p sourced from MSCs alleviates myocardial infarction by targeting pro-apoptotic proteins and EZH2 [183]. In the IR injury model, exosomes derived from MSCs protect cardiac function and reduce infarct size [184]. In addition, the intravenous administration of MSC-derived exosomes reduced infarct size by an impressive 45% and suppressed systemic inflammation [185]. These findings indicate that the specific proteins, peptides, or NcRNAs encapsulated in exosomes, aimed at mitigating myocardial infarction, predominantly target the compromised region. Such intervention modifies the adverse trajectory of myocardial infarction development, underscoring the robust therapeutic efficacy of exosomes. Evaluation of the molecular heterogeneity of exosomes proves to be of paramount importance for the clinical application and for advancing the development of therapeutic vectors.

### 3.3. Exosomes in Heart Failure

Heart failure represents the terminal phase of various CVD. It is characterized by the inability of the heart to supply requisite blood and oxygen to peripheral tissues, fulfilling their metabolic demands [186]. This condition is marked by a series of pathological processes, including cardiomyocyte hypertrophy, cardiac fibrosis, and impaired myocardial angiogenesis [187]. CHD stands as a significant contributor to chronic heart failure [188], and cardiogenic shock is a primary symptom of acute heart failure [189]. Notwithstanding the application of contemporary therapeutic approaches, the mortality and re-admission rates for heart failure remain alarming high. Regrettably, no specific medication exists that can effectively counteract heart failure. Therefore, investigating the pathogenesis of heart failure and pinpointing molecules integral to its onset and progression are imperative for its early diagnosis, effective treatment, and the mitigation of associated complications.

Cardiac hypertrophy is a key mechanism underlying heart failure, and a series of miRNAs have been identified as regulators of cardiac hypertrophy pathogenesis [190]. However, there remains a paucity of analyses concerning the specific components and origins of miRNAs associated with cardiac hypertrophy regulation. This gap in understanding can potentially be bridged by examining exosomal miRNAs (Figure 4C). Under conditions favoring hypertrophy, exosomal miRNAs sourced from diverse cardiovascular cells target cardiomyocytes either to induce or inhibit cardiac hypertrophy. The origin of these exosomal miRNAs suggests three primary mechanisms of action: (1) Intra-cardiomyocyte communication: There is a notable reduction in exosomal miR-133a levels with cardiomyocytes located in the infarcted and surrounding areas in myocardial infarction model [191]. When released from ischemic cardiomyocytes, exosomal miR-133a may be captured by neighboring cardiomyocytes, mitigating hypertrophy by exerting inhibitory effects on cardiomyocyte necrosis and apoptosis. (2) Interplay between cardiac fibroblasts and cardiomyocytes: Cardiac fibroblasts comprise approximately 60–70% of the cardiomyocyte population. Co-culturing cardiomyocytes with fibroblast-conditioned medium has been shown to induce cardiomyocyte hypertrophy, underscoring a pivotal communicative role between the two cell types [192,193]. Cardiac fibroblast-derived exosomes are rich in miR-21-3p, which, when incorporated by cardiomyocytes, downregulates SORBS2 and PDLIM5, thereby facilitating cardiac hypertrophy [29]. (3) Communication with other cardiovascular cell types: Macrophage-derived exosomes enriched in miR-155 promote cardiac hypertrophy and fibrosis in uremic mice by activating the pro-hypertrophic FoxO3a pathway [194]. A recent investigation highlighted that PPAR-γ activation in adipocytes, originating from adipose tissue, augments the secretion of miR-200a-enriched exosomes. This, in turn, promotes cardiac hypertrophy via the mTOR pathway [195]. The intricate interplay of miRNAs, specifically their information exchange and gene regulatory roles between cardiomyocytes and other cardiovascular cells, establishes a multifaceted and dynamic network. This network crucially influences the pathogenesis and progression of cardiac hypertrophy.

Cardiac fibrosis, another important mechanism underlying heart failure, is marked by the degradation of the extracellular matrix and an accumulation of collagen, resultant from the proliferation of cardiac fibroblasts. Numerous studies have elucidated that NcRNAs play a role in the modulation of cardiac fibrosis [196,197]. Mechanically speaking, exosomal NcRNAs, hailing from a plethora of sources, contribute to the intricate regulation of various facets of cardiac fibrosis (Figure 4C). Owing to their efficacy in carrying NcRNAs, exosomes have garnered increased attention for their role in regulating cardiac fibrosis. Throughout the progression of cardiac fibrosis, an upregulation of miR-208a is observed in cardiomyocytes and their derived exosomes. This upregulation facilitates an augmented proliferation of fibroblasts and their subsequent differentiation into myofibroblasts, expediting the progression of cardiac fibrosis [198]. Exosomes that contain LINC00636 serve to inhibit MAPK1 by overexpressing miR-450a-2-3p in human pericardial fluid, thereby ameliorating cardiac fibrosis [199]. Exosome-carried miR-29a, noted for its elevated presence in the marginal ischemic zone, acts as a conduit of information between cardiomyocytes, facilitating anti-fibrotic effects and precluding ventricular dysfunction and heart failure [200]. Exosomal miR-294, derived from embryonic stem cells, has shown its capability to inhibit fibrosis after myocardial infarction, thereby preventing myocardial infarction-induced heart failure [201]. Furthermore, miR-155, found within macrophage-derived exosomes, inhibits the proliferation of cardiac fibroblasts after myocardial infarction in mice, achieving this by targeting and inhibiting SOS-1, a principal regulator of RAS activation [202]. Collectively, these findings underscore the notion that a variety of cell-derived exosomal NcRNAs can either exacerbate or mitigate cardiac fibrosis.

In addition to pathological changes in cardiac tissues, dysregulated angiogenesis, characterized by increased capillary angiogenesis and reduced energy supply to cardiomyocytes, expediently augments the trajectory towards compensatory cardiac hypertrophy and, subsequently, heart failure [203]. The proliferation of ECs stands as a cardinal contributor to this process [204]. Recent findings spotlight the role of exosomal miRNAs in modulating angiogenesis (Figure 4C). Specifically, exosomal miR-21-5-p, derived from healthy hearts, targets and represses PTEN and BCL2, concurrently activating AKT and VEGF pathways in cardiomyocytes and ECs, endorsing both cardiomyocyte proliferation and angiogenesis [205]. During myocardial infarction, M1-like macrophages release a large amount of proinflammatory exosomes that transfer miR-155 to ECs. This action culminates in the downregulation of Rac family small GTPase 1 and p21-activated kinase 2, effectively inhibiting angiogenesis [206]. Notably, the same miRNA can manifest diametrically opposite regulatory behaviors during angiogenesis in heart failure. In instance of cardiomyopathy, exosomes enriched with miR-146a, originating from ECs, significantly attenuate EC proliferation, inhibit microvascular regeneration, and deteriorate both systolic/diastolic function [207]. Contrarily, exosomal miR-146a derived from cardiosphere cells promotes myocardial angiogenesis and significantly ameliorates heart failure subsequent to acute myocardial infarction [208]. These apparent inconsistencies might pertain to the divergent phases of heart failure. While early-stage cardiac exosomes deliver cardioprotective miRNAs, those in the terminal phase may confer detrimental effects, underpinning the multifaceted nature of exosome functionality [209]. The genesis of these contrasting outcomes might be ascribable to the distinct origins of miRNAs coupled with the variable etiologies of heart failure. Further mechanistic investigations are imperative to elucidate these seeming contradictions. Thus, current evidence suggests that exosomal miRNAs exert a bidirectional influence on heart failure, modulated by a spectrum of etiologies and pathological phases.

## 4. Exosomes in Other Cardiomyopathy

### 4.1. Exosomes in Septic Cardiomyopathy

**Figure 5:**
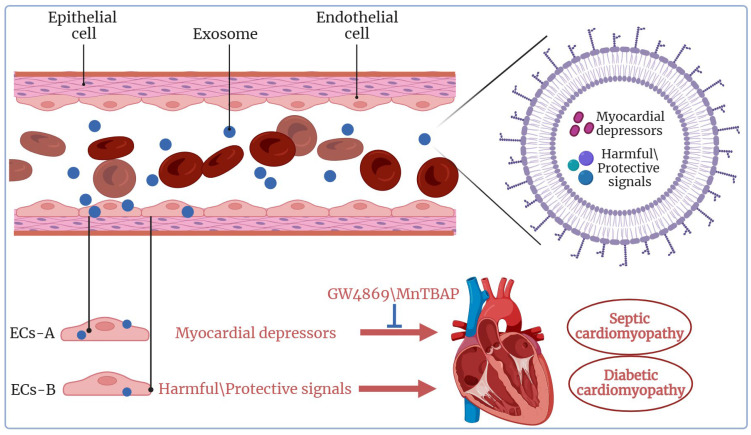
Overview of exosomes in other cardiomyopathy and the underlying mechanisms. Exosomes from cardiomyocytes of sepsis patients contain that can be transmitted to endothelial cells, contributing to the development of septic cardiomyopathy. Promising therapeutic strategies aimed at either inhibiting exosome production (e.g., using GW4869) or eliminating ROS within exosomes (by utilizing MnTBAP) may hold significant potential for patients with septic shock (for detailed information, please refer to the main text). Exosomes released by both diabetic and healthy cardiomyocytes, or those subjected to beneficial modifications under diabetic conditions, possess the capacity to either promote or inhibit the progression of diabetic cardiomyopathy. This figure was created with BioRender.com.

Sepsis is a severe inflammatory response syndrome caused by infection and remains a major contributor to the mortality in the intensive care unit (ICU), characterized by multi-organ dysfunction [210,211]. Cardiovascular dysfunction is a major factor that leads to sepsis-related death [212]. Numerous studies have established a link between sepsis and cardiovascular complications, such as biventricular dilatation and reduced ejection fraction [213], which can lead to CVD and increased mortality rates. Patients with septic cardiomyopathy abnormalities face a mortality rate approximately 2–3 times higher than those without myocardial dysfunction [214,215]. Previous studies have revealed that around 40% of septic patients [213] experience myocardial inhibition [216], with cytokines (IL-β, IL-6, and TNF-α) and reactive oxygen species (ROS)/reactive nitrogen species (RNS) (including nitric oxide (NO), superoxide, and peroxynitrite) contributing to myocardial depression [217,218,219]. Moreover, the presence of exosomes has been implicated in the progression of pathological inflammation (Figure 5) [220,221]. Research has shown that exosomes in sepsis elevated levels of NADPH oxidase, nitric oxide synthase (NOS), and protein disulfide isomerase (PDI) compared to healthy exosomes. In sepsis, both increased NO production and the presence of LPS can trigger the release of platelet exosomes [35]. NO-induced and septic-platelet-derived exosomes induce caspase-3 activation and apoptosis in target ECs by producing active ROS/RNS via NADPH oxidase and NOS type II. Importantly, the neutral sphingomyelinase inhibitor GW4869 is a widely used pharmacological agent to block exosome production [222,223,224]. GW4869 inhibits the ceramide-mediated inward budding of MVBs and the release of mature exosomes from MVBs [97]. Preconditioning with GW4869 can block the production of septic exosomes, significantly reducing the release of sepsis-induced exosomes and pro-inflammatory cytokines, thereby improving cardiac function and survival [225].

Furthermore, endogenous ROS production by exosomes collected from platelets of septic patients induces vascular cell apoptosis [35,226]. In the mouse model of sepsis, circulating exosomes stimulate the formation of podosome clusters in cardiac ECs, leading to increase in vitro and in vivo permeability and cardiac dysfunction [34]. Mechanically, septic exosomes contain higher levels of ROS than normal exosomes. When these ROS are delivered to ECs, they induce vascular leakage and cardiac dysfunction [34]. Interestingly, MnTBAP (MnT), a drug with superoxide dismutase (SOD) and catalase-like activity, can act as a cellular osmotic ROS scavenger, effectively reducing ROS levels within septic exosomes and diminishing their ability to promote podosome cluster formation, thereby inhibiting vascular leakage [227,228]. In addition, the MDVs released by THP-1 monocytes stimulated by LPS contain substantial amount of mitochondrial nucleic acids, proteins, and ROS. These MDV can trigger type I interferon and TNF responses in ECs, further enhancing their proinflammatory potential [229]. Interestingly, these MDVs contain a small amount of exosome-labeled proteins [229], providing compelling evidence that exosomes contribute to inflammation and the development of CVD. Therefore, these findings suggest that exosomes have the potential to induce septic cardiomyopathy, and strategies such as inhibiting exosome production or eliminating ROS within exosomes hold promise as valuable therapeutic approaches for patients with septic shock.

### 4.2. Exosomes in Diabetic Cardiomyopathy

Diabetes mellitus comprises a group of metabolic diseases characterized by hyperglycemia, resulting from either insufficient insulin production (type 1 diabetes) or impaired insulin action (type 2 diabetes) [230]. It is a well-established risk factor for CVD and heart failure. In the early stage of diabetes, hyperglycemia can lead to endothelial dysfunction and microvascular abnormalities [231,232]. Human diabetic cardiomyopathy is characterized by abnormal diastolic function accompanied by mild impairment of systolic function. Pathologically, diabetic cardiomyopathy is associated with cardiomyocyte hypertrophy, necrosis, apoptosis, and increased interstitial fibrosis [233]. During hyperglycemia, intermediates facilitating communication between cardiomyocytes and ECs play a pivotal role, with exosomes emerging as key mediators in this process (Figure 5) [234]. Exosomes can exert either deleterious or beneficial effects on the myocardium, depending on the physiological state of the exosome-derived cells [235,236]. In the context of diabetic cardiomyopathy, diabetic cardiomyocytes release pathogenic exosomes carrying harmful signals. These signals, such as upregulation of miR-320 and the downregulation of IGF-1, HSP20, and Ets2, are received by neighboring ECs, resulting in impaired angiogenesis and a range of cardiovascular injuries, including ventricular dysfunction, cardiac fibrosis, and cardiomyocyte apoptosis [237]. Additionally, exosomes released by diabetic cardiomyocytes have been shown to have detrimental effects on embryonic development. Studies have revealed that exosomes from the hearts of diabetic pregnant mice can induce significant developmental defects, including congenital heart defects in fetuses. Exosomes extracted from diabetic pregnant mice can traverse the maternal-fetal barrier, and exosomal miRNA sequencing analysis has revealed significant differences in the expression of multiple miRNAs, potentially contributing to a high incidence of fetal malformation [238].

Furthermore, aside from their deleterious role in pathological myocardium, exosomes also play a beneficial role in unstimulated animals, preserving the ability of cardiomyocytes to withstand the effects of diabetes and even reversing some of the damage. A recent study by Davidson et al. showed that plasma exosomes from non-diabetic mice can protect rat hearts from IR injury both in vivo and in vitro [239]. These exosomes activate various signaling pathways, including ERK1/2, P38/MAPK, and the phosphorylation of HSP27, a member of the highly cytoprotective family of HSPs, in diabetic rat cardiomyocytes. This activation promotes the interaction of HSP70 with sarcolemmal Toll-like receptor 4 (TLR4), and protects primary cardiomyocytes from IR injury in vitro [239]. While exosomes-derived from healthy rat or human have consistently exhibited a beneficial role in the heart, this role may be altered in response to stress, such as high glucose. Diabetes and hyperglycemia can impair the cardioprotective signaling role of exosomes. Moreover, HSP20, a member of the heat shock protein (HSP) family, plays an important role in intracellular defense [240]. Myocardial overexpression of HSP20 induces qualitative and quantitative changes in the cargo composition and quantity of exosomes secreted by cardiomyocytes. HSP20 directly interacts with TSG101, a protein involved in exosome production, leading to increased exosome production and functional alteration [241]. In transgenic diabetic mice with cardiac-specific overexpression of HSP20, harmful exosomes released by cardiomyocytes are converted into protective exosomes containing phosphorylated Akt, SOD1, and surviving cell, which are cell-protective proteins. These protective exosomes can be delivered to ECs, thereby restoring cardiac function in hyperglycemic conditions [242]. Importantly, blocking exosome production with GW4869 significantly offset the cardioprotective effect mediated by HSP20 in diabetic mice [241]. Hence, it is evident that exosomes from different cell states or physiological conditions may exert opposite effects on the development of diabetic cardiomyopathy. It will be interesting to continue to delve deeper into the differences in molecular changes in cardiomyocytes between diabetic patients and healthy individuals to determine what mediates the transition of exosomes to harmful or beneficial functions.

## 5. Conclusions and Future Directions

Exosomes present a ubiquitous presence in all biological fluids, secreted by virtually every cell type, rendering them a compelling candidate for minimally invasive liquid biopsies and offering the potential for longitudinal sampling to monitor disease progression. Exosomes possess the remarkable capacity for capturing complex molecular cargo from both extracellular and intracellular sources. Furthermore, the surface proteins on exosomes facilitate their immune capture and enrichment, rendering them valuable for comprehensive multiparameter diagnostic assays. These vesicles function as essential mediator for the transmission of a wide spectrum of signals, ranging from protective to pathological, owing to their intricate biogenesis pathways and unique loading mechanisms that adapt to distinct physiological and pathological conditions. Strategies aimed at transforming the enrichment of NcRNAs in exosome, inhibiting exosome production, or eliminating ROS from exosomes hold promise as effective means to intervene in CVD. The capacity of exosomes to deliver functional substances to diseased cells make them favorable therapeutic vectors at both fundamental and practical levels. Ongoing exploration of exosomes, either in their native form or as vehicles for drug delivery, underscores their potential as therapeutic agents. It is important to acknowledge that not all components within exosomes exhibit discernible biological functions. Therefore, a comprehensive and meticulous characterization, complemented by functional assessments, is imperative to unravel the multifaceted biological roles of exosomes in the context of CVD. The clinical translation of exosomes for CVD diagnosis and treatment holds significant promise, but it is clear that there is still a considerable distance to cover.

It is noteworthy that the study of exosomes in the field of CVD is still in its nascent stages, characterized by a paucity of relevant investigations. This scarcity may be attributed to a deficiency in knowledge related to exosomes or technical challenges. Hence, in-depth research endeavors are warranted to gain a comprehensively understanding of the biological dimensions of exosome cargo loading, targeting, delivery, as well as to discern the endogenous contents of exosomes. Currently, the mechanisms governing exosome production and the regulatory processes underpinning their roles in CVD are only partially understood. Addressing a myriad of lingering questions emerging challenges is paramount. These include the following: (1) Deciphering the regulatory mechanism governing the production of cardiac exosomes. (2) Identifying specific exosomal molecules involved in intricate intercellular communication during CVD development. (3) Establishing whether alterations in the composition and content of exosome cargo can yield insights for early diagnosis, prediction, and efficacy evaluation in CVD. (4) Evaluating the potential therapeutic utility of cardiac exosomes in CVD management. (5) Developing strategies to modulate exosome production and cargo loading post-CVD onset to mitigate disease progression. (6) Investigating approaches to modify cardiac exosomes in vivo and in vitro settings to enhance their beneficial effects while mitigating deleterious impacts. Conducting further research into cardiac exosomes will undoubtedly facilitate the discovery of clinical biomarkers for CVD, the development of novel therapeutic targets, advancements in CVD diagnosis and prognosis, and the translation of innovative into clinical practice. This progress is pivotal in alleviating the substantial burden imposed by CVD on healthcare systems and patients.

## Data Availability

Not applicable.
